# Bone Turnover Biomarkers and Hip Fracture Patterns in Older Adults: A Retrospective Cohort Study

**DOI:** 10.3390/jcm15093288

**Published:** 2026-04-25

**Authors:** Damian Mifsut, Jorge Baños-Gómez, Javier Hernández-Balada, Vicent Hurtado-Oliver

**Affiliations:** 1 Hospital Universitario Francesc de Borja, 46702 Gandia, Spain; 2 Surgery Department, Universidad Católica de Valencia San Vicente Mártir, 46001 Valencia, Spain; 3 Anathomy Department, Universitat de València, 46022 Valencia, Spain

**Keywords:** hip fracture, bone turnover markers, beta-CTX, PINP, vitamin D, parathyroid hormone, osteoporosis, elderly

## Abstract

**Background:** Hip fractures represent a major public health challenge in aging populations and are associated with high morbidity, mortality, and healthcare costs. While osteoporosis is the main underlying cause, biochemical markers of bone metabolism may provide additional insight into skeletal remodeling processes. However, the relationship between bone turnover biomarkers and specific hip fracture patterns remains poorly understood. **Methods:** A retrospective observational study was conducted, including patients admitted with hip fractures between January 2022 and December 2023 at our institution. Serum levels of vitamin D, parathyroid hormone (PTH), N-terminal propeptide of type I collagen (PINP), and beta-C-terminal telopeptide of type I collagen (β-CTX) were analyzed. Fractures were classified as intracapsular or extracapsular. Continuous variables were compared using the Mann–Whitney U test. Multivariable logistic regression analysis was performed to identify factors independently associated with extracapsular fractures. **Results:** A total of 131 patients were included, comprising 57 intracapsular fractures and 74 extracapsular fractures. Patients with extracapsular fractures were significantly older (83 (75–89) vs. 80 (71–86) years; *p* = 0.0079). No significant differences were observed in vitamin D levels between fracture groups (*p* = 0.446). PTH levels were higher in extracapsular fractures (*p* = 0.030), while β-CTX levels tended to be lower (*p* = 0.080). In multivariable logistic regression analysis, age remained independently associated with extracapsular fracture pattern (OR 1.05, 95% CI 1.01–1.09; *p* = 0.03). Higher β-CTX levels were inversely associated with extracapsular fractures (OR 0.65, 95% CI 0.43–0.96; *p* = 0.03), whereas vitamin D levels were not independently associated with fracture type. **Conclusions:** Extracapsular hip fractures were primarily associated with older age in this cohort. Among bone metabolism biomarkers, β-CTX showed an inverse association with extracapsular fracture pattern after adjustment for confounding factors. These findings should be interpreted with caution and considered exploratory, highlighting the need for prospective studies to clarify their clinical significance.

## 1. Introduction

Hip fractures represent one of the most serious consequences of osteoporosis and constitute a major challenge for healthcare systems worldwide. With the progressive aging of the population, the incidence of hip fractures continues to increase, leading to substantial morbidity, mortality, and healthcare costs [1,2]. Patients with hip fractures often experience long-term disability and loss of independence, highlighting the importance of identifying factors associated with fracture risk and fracture characteristics [3].

Although bone mineral density (BMD) is widely used to assess fracture risk, bone strength is determined by additional factors such as bone quality, microarchitecture, and bone turnover dynamics [4]. Biochemical markers of bone metabolism provide valuable information regarding the balance between bone formation and bone resorption and have been increasingly used to evaluate skeletal remodeling processes [5].

Among these biomarkers, vitamin D and parathyroid hormone (PTH) play central roles in calcium homeostasis and bone metabolism [6,7,8]. Vitamin D deficiency is highly prevalent among elderly individuals and has been associated with increased risk of fragility fractures [6,7]. Parathyroid hormone regulates calcium metabolism and may contribute to increased bone turnover when elevated [8].

In addition, bone turnover markers such as N-terminal propeptide of type I collagen (PINP), a marker of bone formation, and beta-C-terminal telopeptide of type I collagen (β-CTX), a marker of bone resorption, provide insight into skeletal remodeling activity [5]. These markers have been investigated in relation to fracture risk and osteoporosis treatment monitoring [9].

Hip fractures are typically classified into intracapsular fractures, occurring within the joint capsule of the femoral neck, and extracapsular fractures, including intertrochanteric and subtrochanteric fractures [10]. These fracture patterns differ in biomechanical characteristics, vascular supply, and surgical management. However, the potential association between bone turnover biomarkers and hip fracture pattern remains insufficiently understood [11,12].

The aim of this study was to evaluate the relationship between bone metabolism biomarkers and hip fracture type by comparing intracapsular and extracapsular fractures in a cohort of patients treated at a tertiary hospital.

## 2. Materials and Methods

### 2.1. Study Design and Population

A retrospective observational study was conducted at our institution. Patients admitted with a diagnosis of hip fracture between January 2022 and December 2023 were eligible for inclusion.

Inclusion criteria were:

- Age ≥ 50 years;
- Radiologically confirmed hip fracture;
- Availability of biochemical markers of bone metabolism obtained during hospital admission.

Patients with pathological fractures related to malignancy or high-energy trauma were excluded.

### 2.2. Fracture Classification

Hip fractures were classified according to anatomical location into:

- Intracapsular fractures (femoral neck fractures);
- Extracapsular fractures (intertrochanteric fractures).

Fracture classification was performed based on radiographic evaluation.

### 2.3. Biochemical Measurements

Fasting blood samples were obtained in the morning within the first 24 h of hospital admission following the fracture and prior to surgical intervention, and were analyzed for the following biomarkers:25-hydroxyvitamin D

- Parathyroid hormone (PTH);
- N-terminal propeptide of type I collagen (PINP);
- β-C-terminal telopeptide of type I collagen (β-CTX).

These biomarkers were selected as indicators of bone metabolism, reflecting bone formation and bone resorption activity.

In addition to bone turnover markers, routine biochemical parameters related to mineral metabolism were also collected, including serum calcium, corrected calcium, phosphate, total alkaline phosphatase, bone alkaline phosphatase, and estimated glomerular filtration rate (eGFR).

### 2.4. Statistical Analysis

Continuous variables were assessed for normality using the Shapiro–Wilk test. Because biomarker distributions were non-normal, results are presented as medians and interquartile range (IQR). Comparisons between intracapsular and extracapsular fractures were performed using the Mann–Whitney U test.

Multivariable logistic regression analysis was used to evaluate independent associations between biomarkers and extracapsular fracture patterns. Biomarkers with skewed distributions were log-transformed before inclusion in the model. Variables included in the multivariable logistic regression model were selected based on clinical relevance and biological plausibility rather than solely on univariate statistical significance. Particular attention was given to avoiding overfitting and multicollinearity, especially among interrelated variables involved in calcium and bone metabolism (e.g., vitamin D, PTH, and calcium-related parameters).

Prior to model construction, collinearity between candidate predictors was assessed using variance inflation factors (VIFs). Based on these considerations, a parsimonious model was constructed including age, sex, vitamin D, β-CTX, and renal function (eGFR), while avoiding the simultaneous inclusion of multiple highly correlated biochemical variables.

A sensitivity analysis including PTH was considered; however, given the limited sample size and potential collinearity, inclusion of multiple correlated variables was deemed to risk model instability.

Extracapsular fracture pattern was used as the dependent variable in the logistic regression model.

Odds ratios (ORs) with 95% confidence intervals (CI) were calculated. A *p* value < 0.05 was considered statistically significant.

## 3. Results

### 3.1. Patient Characteristics

A total of 131 patients with hip fractures were included in the study. Among them, 57 patients (43.5%) presented intracapsular fractures, whereas 74 patients (56.5%) presented extracapsular fractures.

Patients with extracapsular fractures were significantly older than those with intracapsular fractures (83 (75–89) vs. 80 (71–86) years; *p* = 0.0079).

Women represented the majority of patients in both groups, accounting for 66.7% of intracapsular fractures and 73.0% of extracapsular fractures (*p* = 0.56).

Baseline characteristics of the study population are presented in Table 1.

### 3.2. Bone Metabolism Biomarkers

Serum PTH levels were significantly higher in patients with extracapsular fractures compared with those with intracapsular fractures (*p* = 0.030). Corrected calcium and total alkaline phosphatase levels were also significantly higher in patients with extracapsular fractures (*p* = 0.015 and *p* = 0.016, respectively), suggesting increased bone turnover activity.

No statistically significant differences were observed in vitamin D levels, serum phosphate, bone alkaline phosphatase, renal function, or PINP levels between fracture groups.

**Figure 1:**
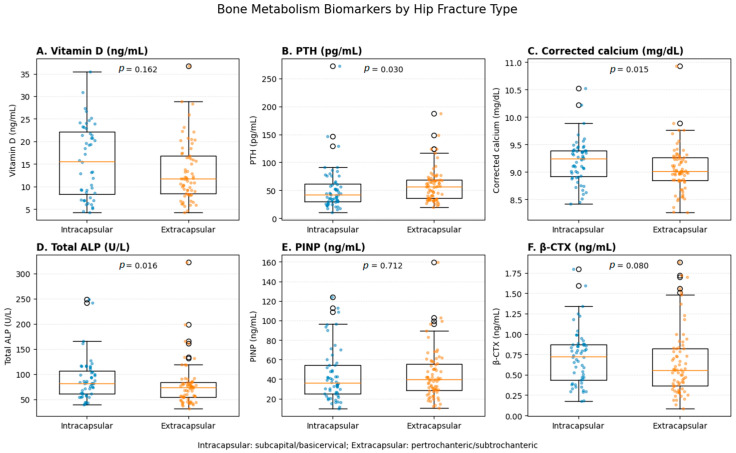
Distribution of bone metabolism biomarkers according to hip fracture type. Boxplots represent the median and interquartile range, with individual data points overlaid. PTH, corrected calcium and total ALP were significantly higher in extracapsular fractures.

β-CTX levels tended to be lower in extracapsular fractures compared with intracapsular fractures, although this difference did not reach statistical significance (*p* = 0.080) (Figure 1).

### 3.3. Multivariable Logistic Regression

Multivariable logistic regression analysis was performed, including age, sex, vitamin D, β-CTX, and estimated glomerular filtration rate (Table 2). Although PTH showed a statistically significant association in the univariate analysis, it was not included in the final multivariable logistic regression model. Variable selection for the multivariable model was based on clinical relevance and potential collinearity between variables related to bone metabolism.

PTH was considered strongly related to vitamin D status and calcium metabolism, and therefore, the inclusion of multiple highly correlated biochemical variables could have introduced multicollinearity and reduced the stability of the model estimates. For this reason, vitamin D and β-CTX were prioritized as representative markers of bone metabolism and bone turnover, while PTH was excluded from the final model.

Before constructing the final model, potential collinearity between predictors was evaluated, particularly among vitamin D, PTH, and calcium-related parameters. This assessment suggested a degree of interdependence between these variables, supporting the decision to avoid including all of them simultaneously in the multivariable model.

The selection of variables for the final logistic regression model was therefore guided by: Clinical relevance, Results of the univariate analysis, and Considerations of multicollinearity among biologically related predictors.

This approach aimed to obtain a parsimonious and statistically stable model while preserving the most clinically meaningful predictors.

Age remained independently associated with extracapsular fracture pattern (OR 1.05, 95% CI 1.01–1.09; *p* = 0.03). Higher β-CTX levels were inversely associated with extracapsular fractures (OR 0.65, 95% CI 0.43–0.96; *p* = 0.03).

## 4. Discussion

Hip fractures represent a major challenge for healthcare systems due to their increasing incidence, high morbidity, and substantial economic burden worldwide [1,2]. Understanding the biological mechanisms underlying different fracture patterns may provide valuable insights into skeletal fragility and potential prevention strategies.

In the present study, extracapsular fractures were primarily associated with older age, which remained an independent predictor after multivariable adjustment. This finding is consistent with previous studies indicating that intertrochanteric fractures occur more frequently in older and more frail patients [12,13]. Age-related changes in bone geometry, cortical thinning, and deterioration of trabecular microarchitecture may contribute to the increased susceptibility to extracapsular fractures in very elderly individuals.

The finding of higher PTH levels in patients with extracapsular fractures in the univariate analysis is also noteworthy. Elevated PTH levels in older adults are commonly associated with secondary hyperparathyroidism, often driven by vitamin D deficiency, reduced calcium intake, and age-related decline in renal function [8,14]. Chronic elevation of PTH may lead to increased bone turnover and preferential cortical bone loss, which could influence fracture patterns, particularly in regions with a higher cortical bone component.

Although PTH was not included in the final multivariable model due to concerns regarding collinearity with other calcium metabolism variables, this finding remains clinically relevant and suggests that disturbances in calcium–PTH–vitamin D homeostasis may contribute to skeletal fragility in specific fracture types. Further studies are needed to better clarify this relationship.

Interestingly, β-CTX showed an inverse association with extracapsular fractures after multivariable adjustment. β-CTX is a marker of osteoclastic bone resorption and reflects systemic skeletal turnover [5,15,16,17]. Although higher bone turnover is generally associated with increased fracture risk, the relationship between bone resorption markers and specific fracture morphology remains poorly understood. One possible explanation is that acute fracture events and trauma-related metabolic responses may alter circulating levels of bone turnover markers, thereby affecting their interpretation in the acute setting.

Although β-CTX did not reach statistical significance in the univariate analysis (*p* = 0.080), it emerged as a significant predictor in the multivariable logistic regression model. This discrepancy may be explained by the presence of confounding variables, particularly age and other biochemical parameters related to bone metabolism, which may influence both fracture pattern and bone turnover markers.

In the univariate analysis, the effect of β-CTX may have been partially masked by these confounding factors. After adjusting for potential confounders in the multivariable model, the independent association between β-CTX levels and fracture type became evident. This phenomenon is commonly observed when variables are interrelated, and adjustment allows the independent contribution of a predictor to be more accurately estimated. The observation that β-CTX did not reach statistical significance in the univariate analysis but became significant after multivariable adjustment likely reflects the influence of confounding factors. In particular, age and renal function are known to affect both bone turnover markers and fracture characteristics. In our cohort, adjustment for these variables may have unmasked the independent association between β-CTX and fracture pattern.

From a biological perspective, lower β-CTX levels in patients with extracapsular fractures may reflect different underlying skeletal states. One possible explanation is that reduced bone turnover could be associated with impaired bone remodeling capacity and accumulation of microdamage, leading to increased structural fragility. Alternatively, given that biomarker measurements were obtained during the acute post-fracture period, lower β-CTX levels may also be influenced by acute-phase responses to trauma, inflammation, or immobilization. Therefore, this finding should be interpreted cautiously, as it may not represent baseline bone metabolism.

Additionally, potential collinearity between variables related to bone metabolism (e.g., vitamin D, PTH, and bone turnover markers) was assessed before model construction. Variance inflation factors (VIFs) were examined and did not indicate significant multicollinearity, suggesting that the predictors included in the model contributed independently to the outcome.

Taken together, these findings suggest that β-CTX may represent an independent marker associated with fracture type once relevant confounding factors are taken into account.

An important methodological consideration is that biomarker measurements were obtained shortly after the fracture event. Previous studies have shown that bone turnover markers may change acutely following fracture, potentially reflecting trauma-related metabolic responses rather than baseline skeletal physiology. Therefore, the associations observed in this study should be interpreted cautiously, as they may partially reflect acute-phase alterations rather than pre-fracture bone metabolism.

Recent studies have highlighted the complex relationship between bone metabolism and hip fracture characteristics. Large cohort studies have shown that while bone turnover markers such as PINP and β-CTX are useful for predicting overall fracture risk and monitoring osteoporosis treatment, their ability to discriminate between different fracture patterns remains limited [9,18]. These associations are often influenced by multiple confounding factors, including age, renal function, nutritional status, and frailty.

Another important consideration is that biochemical measurements obtained during acute hospitalization may not fully reflect baseline skeletal metabolism. Trauma-related inflammatory responses, immobilization, and perioperative metabolic changes may influence biomarker concentrations [19]. Consequently, the interpretation of bone turnover markers in the context of acute fractures should be performed with caution.

Overall, our findings suggest that age-related skeletal fragility may play a more prominent role than systemic bone turnover activity in determining hip fracture patterns. Extracapsular fractures may therefore reflect a combination of advanced age, biomechanical vulnerability, and structural changes in the proximal femur rather than a purely metabolic high-turnover state.

### 4.1. Clinical Implications

The identification of metabolic and demographic factors associated with different hip fracture patterns may contribute to improved risk stratification in older adults. Although bone turnover biomarkers did not strongly discriminate fracture types in our cohort, their evaluation may still provide useful information regarding skeletal metabolism and potential therapeutic strategies in patients with fragility fractures.

### 4.2. Limitations

Several limitations should be acknowledged. First, the retrospective design precludes causal inference. Second, biochemical measurements were obtained within the first 24 h after hospital admission and therefore may have been influenced by acute fracture-related metabolic and inflammatory responses. As a result, these biomarker levels may not accurately reflect baseline bone metabolism and may introduce bias in the observed associations.

Third, precise data regarding the time interval between fracture occurrence and blood sampling were not systematically available, which limits the ability to assess the impact of timing on biomarker levels.

Fourth, the relatively small sample size may have limited statistical power and contributed to instability in multivariable estimates.

Finally, important confounding variables such as bone mineral density, prior osteoporosis treatment, and nutritional status were not available and may have influenced both biomarker levels and fracture patterns.

## 5. Conclusions

In this retrospective cohort of patients with hip fractures, extracapsular fractures were primarily associated with older age. Among bone metabolism biomarkers, PTH levels were higher in extracapsular fractures in unadjusted analyses, whereas β-CTX showed an inverse association with extracapsular fracture pattern after multivariable adjustment.

However, these findings should be interpreted with caution. The retrospective design, the relatively small sample size, and the measurement of biomarkers during the acute post-fracture period may limit causal inference and introduce bias related to trauma-induced metabolic changes.

Overall, our results suggest that age remains the main clinical factor associated with hip fracture pattern, while bone metabolism biomarkers such as β-CTX and PTH may provide additional pathophysiological insight but have limited predictive value for fracture type.

These findings should therefore be considered exploratory and hypothesis-generating. Further prospective studies with standardized biomarker assessment and comprehensive adjustment for confounders are needed to clarify the relationship between bone metabolism and hip fracture morphology.

## Figures and Tables

**Table 1:** **Baseline characteristics and biochemical parameters according to hip fracture type.** Data are presented as medians (IQR). Comparisons between groups were performed using the Mann–Whitney U test. Intracapsular fractures include femoral neck fractures, whereas extracapsular fractures include pertrochanteric fractures.

Variable	Intracapsular (*n* = 57)	Extracapsular (*n* = 74)	*p*-Value
Age, years	80 (71–86)	83 (75–89)	0.0079
Vitamin D, ng/mL	14 (8–22)	12 (7–19)	0.162
PTH, pg/mL	38 (24–60)	52 (30–79)	0.030
Calcium, mg/dL	9.2 (8.8–9.5)	9.2 (8.8–9.6)	0.080
Corrected calcium, mg/dL	9.3 (9.0–9.6)	9.4 (9.0–9.7)	0.015
Phosphate, mg/dL	3.4 (2.9–3.9)	3.5 (3.0–3.9)	0.461
Total ALP, U/L	90 (69–116)	95 (70–126)	0.016
Bone ALP, U/L	16 (11–23)	18 (12–26)	0.792
eGFR, mL/min/1.73 m^2^	60 (43–77)	57 (39–74)	0.530
PINP, ng/mL	35 (23–54)	38 (24–56)	0.712
β-CTX, ng/mL	0.67 (0.47–0.88)	0.59 (0.39–0.80)	0.080

**Table 2:** **Multivariable logistic regression analysis of factors associated with hip fracture type**. Odds ratios (OR) with 95% confidence intervals (CI) are shown for age, β-CTX, vitamin D, sex, and estimated glomerular filtration rate (eGFR). Age was significantly associated with fracture type (OR = 1.05; 95% CI: 1.01–1.09; *p* = 0.03), indicating an increased likelihood of the outcome with advancing age. β-CTX levels were also significantly associated with fracture type (OR = 0.65; 95% CI: 0.43–0.96; *p* = 0.03). In contrast, vitamin D (OR = 0.98; 95% CI: 0.94–1.03; *p* = 0.31), sex (OR = 0.85; 95% CI: 0.40–1.80; *p* = 0.68), and eGFR (OR = 0.99; 95% CI: 0.97–1.01; *p* = 0.41) were not significantly associated with fracture type.

Variable	OR	95% CI	*p*
Age	1.05	1.01–1.09	0.03
Β-CTX	0.65	0.43–0.96	0.003
Vitamin D	0.98	0.94–1.03	0.31
Sex	0.85	0.40–1.80	0.68
eGFR	0.99	0.97–1.01	0.41

## Data Availability

The datasets generated and analyzed during the current study are available from the corresponding author upon reasonable request.
